# Supplementary material for: Unveiling the Bioactive Potential of the Invasive Jellyfish Phyllorhiza punctata Through Integrative Transcriptomic and Proteomic Analyses
Source: Biomolecules. 2025 Aug 4;15(8):1121. doi: 10.3390/biom15081121 (PMC12383608; doi:10.3390/biom15081121)
Supplement: Supplementary file 1 [file biomolecules-15-01121-s001.zip › Suplementary Material/FileS3/PEPFOLD4/XP_065067357_14265/clusters.html]

 PEP-FOLD clusters


Download archive of all models  here   

|  |  |  |
| --- | --- | --- |
| # | file | sOPEP |
| # cluster 1 - Size: 3 |  |  |
|  | XP\_065067357\_14265-model1 | -36.0747 |
|  | XP\_065067357\_14265-model1.1 | -33.9683 |
|  | XP\_065067357\_14265-model1.2 | -21.588 |
| # cluster 2 - Size: 5 |  |  |
|  | XP\_065067357\_14265-model2 | -35.8614 |
|  | XP\_065067357\_14265-model2.1 | -33.8648 |
|  | XP\_065067357\_14265-model2.2 | -33.7677 |
|  | XP\_065067357\_14265-model2.3 | -21.7854 |
|  | XP\_065067357\_14265-model2.4 | -19.0155 |
| # cluster 3 - Size: 1 |  |  |
|  | XP\_065067357\_14265-model3 | -35.3047 |
| # cluster 4 - Size: 1 |  |  |
|  | XP\_065067357\_14265-model4 | -35.2662 |
| # cluster 5 - Size: 3 |  |  |
|  | XP\_065067357\_14265-model5 | -34.3984 |
|  | XP\_065067357\_14265-model5.1 | -32.3545 |
|  | XP\_065067357\_14265-model5.2 | -25.5421 |
| # cluster 6 - Size: 3 |  |  |
|  | XP\_065067357\_14265-model6 | -33.8806 |
|  | XP\_065067357\_14265-model6.1 | -28.3294 |
|  | XP\_065067357\_14265-model6.2 | -23.3898 |
| # cluster 7 - Size: 1 |  |  |
|  | XP\_065067357\_14265-model7 | -32.8798 |
| # cluster 8 - Size: 1 |  |  |
|  | XP\_065067357\_14265-model8 | -32.6735 |
| # cluster 9 - Size: 6 |  |  |
|  | XP\_065067357\_14265-model9 | -32.2405 |
|  | XP\_065067357\_14265-model9.1 | -31.1112 |
|  | XP\_065067357\_14265-model9.2 | -28.828 |
|  | XP\_065067357\_14265-model9.3 | -23.8101 |
|  | XP\_065067357\_14265-model9.4 | -22.0919 |
|  | XP\_065067357\_14265-model9.5 | -19.6014 |
| # cluster 10 - Size: 1 |  |  |
|  | XP\_065067357\_14265-model10 | -32.2224 |
| # cluster 11 - Size: 1 |  |  |
|  | XP\_065067357\_14265-model11 | -31.9189 |
| # cluster 12 - Size: 1 |  |  |
|  | XP\_065067357\_14265-model12 | -31.7127 |
| # cluster 13 - Size: 1 |  |  |
|  | XP\_065067357\_14265-model13 | -31.3217 |
| # cluster 14 - Size: 1 |  |  |
|  | XP\_065067357\_14265-model14 | -31.0486 |
| # cluster 15 - Size: 2 |  |  |
|  | XP\_065067357\_14265-model15 | -30.9444 |
|  | XP\_065067357\_14265-model15.1 | -28.314 |
| # cluster 16 - Size: 1 |  |  |
|  | XP\_065067357\_14265-model16 | -30.5073 |
| # cluster 17 - Size: 1 |  |  |
|  | XP\_065067357\_14265-model17 | -30.3014 |
| # cluster 18 - Size: 2 |  |  |
|  | XP\_065067357\_14265-model18 | -29.2773 |
|  | XP\_065067357\_14265-model18.1 | -25.2247 |
| # cluster 19 - Size: 1 |  |  |
|  | XP\_065067357\_14265-model19 | -29.2303 |
| # cluster 20 - Size: 1 |  |  |
|  | XP\_065067357\_14265-model20 | -29.0185 |
| # cluster 21 - Size: 1 |  |  |
|  | XP\_065067357\_14265-model21 | -28.9378 |
| # cluster 22 - Size: 1 |  |  |
|  | XP\_065067357\_14265-model22 | -28.705 |
| # cluster 23 - Size: 6 |  |  |
|  | XP\_065067357\_14265-model23 | -28.6082 |
|  | XP\_065067357\_14265-model23.1 | -27.7241 |
|  | XP\_065067357\_14265-model23.2 | -26.0519 |
|  | XP\_065067357\_14265-model23.3 | -23.75 |
|  | XP\_065067357\_14265-model23.4 | -22.9101 |
|  | XP\_065067357\_14265-model23.5 | -20.7743 |
| # cluster 24 - Size: 4 |  |  |
|  | XP\_065067357\_14265-model24 | -27.9082 |
|  | XP\_065067357\_14265-model24.1 | -27.1756 |
|  | XP\_065067357\_14265-model24.2 | -26.7862 |
|  | XP\_065067357\_14265-model24.3 | -23.2979 |
| # cluster 25 - Size: 3 |  |  |
|  | XP\_065067357\_14265-model25 | -27.1628 |
|  | XP\_065067357\_14265-model25.1 | -23.4271 |
|  | XP\_065067357\_14265-model25.2 | -23.2841 |
| # cluster 26 - Size: 2 |  |  |
|  | XP\_065067357\_14265-model26 | -26.7996 |
|  | XP\_065067357\_14265-model26.1 | -22.718 |
| # cluster 27 - Size: 1 |  |  |
|  | XP\_065067357\_14265-model27 | -26.5187 |
| # cluster 28 - Size: 3 |  |  |
|  | XP\_065067357\_14265-model28 | -26.4431 |
|  | XP\_065067357\_14265-model28.1 | -25.8944 |
|  | XP\_065067357\_14265-model28.2 | -19.7735 |
| # cluster 29 - Size: 1 |  |  |
|  | XP\_065067357\_14265-model29 | -25.9607 |
| # cluster 30 - Size: 1 |  |  |
|  | XP\_065067357\_14265-model30 | -25.8476 |
| # cluster 31 - Size: 2 |  |  |
|  | XP\_065067357\_14265-model31 | -24.9652 |
|  | XP\_065067357\_14265-model31.1 | -22.9982 |
| # cluster 32 - Size: 1 |  |  |
|  | XP\_065067357\_14265-model32 | -24.8261 |
| # cluster 33 - Size: 3 |  |  |
|  | XP\_065067357\_14265-model33 | -24.5798 |
|  | XP\_065067357\_14265-model33.1 | -23.3958 |
|  | XP\_065067357\_14265-model33.2 | -21.7164 |
| # cluster 34 - Size: 1 |  |  |
|  | XP\_065067357\_14265-model34 | -24.4922 |
| # cluster 35 - Size: 1 |  |  |
|  | XP\_065067357\_14265-model35 | -24.0815 |
| # cluster 36 - Size: 2 |  |  |
|  | XP\_065067357\_14265-model36 | -24.0066 |
|  | XP\_065067357\_14265-model36.1 | -22.5874 |
| # cluster 37 - Size: 2 |  |  |
|  | XP\_065067357\_14265-model37 | -23.8254 |
|  | XP\_065067357\_14265-model37.1 | -20.1265 |
| # cluster 38 - Size: 1 |  |  |
|  | XP\_065067357\_14265-model38 | -23.7775 |
| # cluster 39 - Size: 1 |  |  |
|  | XP\_065067357\_14265-model39 | -23.2359 |
| # cluster 40 - Size: 1 |  |  |
|  | XP\_065067357\_14265-model40 | -23.1388 |
| # cluster 41 - Size: 1 |  |  |
|  | XP\_065067357\_14265-model41 | -22.9514 |
| # cluster 42 - Size: 1 |  |  |
|  | XP\_065067357\_14265-model42 | -22.8937 |
| # cluster 43 - Size: 1 |  |  |
|  | XP\_065067357\_14265-model43 | -22.6033 |
| # cluster 44 - Size: 2 |  |  |
|  | XP\_065067357\_14265-model44 | -22.0061 |
|  | XP\_065067357\_14265-model44.1 | -19.6287 |
| # cluster 45 - Size: 1 |  |  |
|  | XP\_065067357\_14265-model45 | -21.8781 |
| # cluster 46 - Size: 1 |  |  |
|  | XP\_065067357\_14265-model46 | -21.6969 |
| # cluster 47 - Size: 1 |  |  |
|  | XP\_065067357\_14265-model47 | -21.3925 |
| # cluster 48 - Size: 1 |  |  |
|  | XP\_065067357\_14265-model48 | -21.2882 |
| # cluster 49 - Size: 1 |  |  |
|  | XP\_065067357\_14265-model49 | -21.1018 |
| # cluster 50 - Size: 1 |  |  |
|  | XP\_065067357\_14265-model50 | -20.9474 |
| # cluster 51 - Size: 1 |  |  |
|  | XP\_065067357\_14265-model51 | -20.6881 |
| # cluster 52 - Size: 1 |  |  |
|  | XP\_065067357\_14265-model52 | -20.6437 |
| # cluster 53 - Size: 1 |  |  |
|  | XP\_065067357\_14265-model53 | -20.4212 |
| # cluster 54 - Size: 1 |  |  |
|  | XP\_065067357\_14265-model54 | -20.3606 |
| # cluster 55 - Size: 1 |  |  |
|  | XP\_065067357\_14265-model55 | -20.1968 |
| # cluster 56 - Size: 1 |  |  |
|  | XP\_065067357\_14265-model56 | -20.1807 |
| # cluster 57 - Size: 1 |  |  |
|  | XP\_065067357\_14265-model57 | -20.1089 |
| # cluster 58 - Size: 1 |  |  |
|  | XP\_065067357\_14265-model58 | -19.7228 |
| # cluster 59 - Size: 1 |  |  |
|  | XP\_065067357\_14265-model59 | -19.5738 |
| # cluster 60 - Size: 1 |  |  |
|  | XP\_065067357\_14265-model60 | -19.5119 |
| # cluster 61 - Size: 1 |  |  |
|  | XP\_065067357\_14265-model61 | -19.3451 |
| # cluster 62 - Size: 1 |  |  |
|  | XP\_065067357\_14265-model62 | -18.775 |
| # cluster 63 - Size: 1 |  |  |
|  | XP\_065067357\_14265-model63 | -18.6447 |
| # cluster 64 - Size: 1 |  |  |
|  | XP\_065067357\_14265-model64 | -17.1378 |
|  |  |  |
